# Identification of Transcripts with Shared Roles in the Pathogenesis of Postmenopausal Osteoporosis and Cardiovascular Disease

**DOI:** 10.3390/ijms25105554

**Published:** 2024-05-20

**Authors:** Sjur Reppe, Sveinung Gundersen, Geir K. Sandve, Yunpeng Wang, Ole A. Andreassen, Carolina Medina-Gomez, Fernando Rivadeneira, Tor P. Utheim, Eivind Hovig, Kaare M. Gautvik

**Affiliations:** 1 Department of Medical Biochemistry, Oslo University Hospital, 0450 Oslo, Norway; 2 Unger-Vetlesen Institute, Lovisenberg Diaconal Hospital, 0440 Oslo, Norway; 3 Department of Plastic and Reconstructive Surgery, Oslo University Hospital, 0424 Oslo, Norway; 4 Center for Bioinformatics, Department of Informatics, University of Oslo, 0313 Oslo, Norway; 5 Department of Informatics, University of Oslo, 0373 Oslo, Norway; geirksa@ifi.uio.no (G.K.S.);; 6 NORMENT, Institute of Clinical Medicine, University of Oslo, 0450 Oslo, Norway; yunpeng.wang@psykologi.uio.no (Y.W.); o.a.andreassen@medisin.uio.no (O.A.A.); 7 Division of Mental Health and Addiction, Oslo University Hospital, 0424 Oslo, Norway; 8 Department of Neurosciences, University of California San Diego, La Jolla, CA 92093, USA; 9 Department of Psychiatry, University of California San Diego, La Jolla, CA 92093, USA; 10 Department of Internal Medicine, Erasmus MC, University Medical Center Rotterdam, 3015 GD Rotterdam, The Netherlands; m.medinagomez@erasmusmc.nl (C.M.-G.); f.rivadeneira@erasmusmc.nl (F.R.); 11 Department of Tumor Biology, Institute for Cancer Research, Oslo University Hospital, 0424 Oslo, Norway

**Keywords:** cardiovascular disease, osteoporosis, transcriptomes, genes, pleiotropic

## Abstract

Epidemiological evidence suggests existing comorbidity between postmenopausal osteoporosis (OP) and cardiovascular disease (CVD), but identification of possible shared genes is lacking. The skeletal global transcriptomes were analyzed in trans-iliac bone biopsies (n = 84) from clinically well-characterized postmenopausal women (50 to 86 years) without clinical CVD using microchips and RNA sequencing. One thousand transcripts highly correlated with areal bone mineral density (aBMD) were further analyzed using bioinformatics, and common genes overlapping with CVD and associated biological mechanisms, pathways and functions were identified. Fifty genes (45 mRNAs, 5 miRNAs) were discovered with established roles in oxidative stress, inflammatory response, endothelial function, fibrosis, dyslipidemia and osteoblastogenesis/calcification. These pleiotropic genes with possible CVD comorbidity functions were also present in transcriptomes of microvascular endothelial cells and cardiomyocytes and were differentially expressed between healthy and osteoporotic women with fragility fractures. The results were supported by a genetic pleiotropy-informed conditional False Discovery Rate approach identifying any overlap in single nucleotide polymorphisms (SNPs) within several genes encoding aBMD- and CVD-associated transcripts. The study provides transcriptional and genomic evidence for genes of importance for both BMD regulation and CVD risk in a large collection of postmenopausal bone biopsies. Most of the transcripts identified in the CVD risk categories have no previously recognized roles in OP pathogenesis and provide novel avenues for exploring the mechanistic basis for the biological association between CVD and OP.

## 1. Introduction

Cardiovascular disease (CVD) and osteoporosis (OP) comprise two of the most common age-related chronic conditions and their prevalence is increasing [1,2]. The rise in absolute number and longevity of elderly will lead to a further increase in morbidity and mortality due to osteoporotic fragility fractures and CVD. Of note, women with OP have a four-fold increase in risk for an acute CVD event [3]. The relationship between the two conditions persists even after adjustment for potential confounding risk factors, and shared inherited risk between these two age-related chronic diseases has therefore been suggested [4].

OP is a polygenic disorder, and genetic factors have been shown to have an important role in regulating bone mineral density (BMD) and the risk of low-energy osteoporotic fractures [5,6]. Also, different CVD traits ranging from congenital to ischemic heart disease to peripheral arterial illnesses have heritable components [7]. Our previous identification of 65 loci common to both CVD-associated features and BMD levels supports the notion of shared risk genes [6] and prompted us to a more in depth functional genetic analysis.

Several studies have attempted to identify common underlying pathophysiological processes and their respective mediators in order to explain the established link between CVD and OP [8,9,10,11]. A common feature of both diseases appears to be a low-grade chronic state of inflammation which paradoxically promotes bone formation/calcification of the vasculature [12], but also induces decalcification of hard tissue [13]. The calcification occurs both by deposition of calcium and phosphate on elastin and collagen fibrils in vessels via a passive mechanism independent of cellular activity and also by an active process involving inflammation preceding calcification [13]. An inflammatory reaction is promoted by lipid oxidation [8] and leads to stimulation of the RANK/RANKL/OPG system promoting vascular calcification [14] as well as activation and differentiation of bone-resorbing osteoclasts [15]. Another common signaling system involved in vascular calcification and bone metabolism is the Wnt-β-catenin pathway comprising, e.g., sclerostin and Dkk1 proteins as important inhibitors, and their circulating levels have been shown to be inversely related to arterial calcifications [16,17]. In bone biopsies from postmenopausal women, sclerostin (*SOST*) and *DKK1* mRNAs show a strong association to BMD levels [18,19]. Evidence of a possible link between OP and CVD involving Wnt signaling is also supported by family studies, e.g., a *LRP6* mutation resulting in a Wnt signaling defect occurs in a family with autosomal dominant early CVD with features of metabolic syndrome, and osteoporosis [20].

To identify possible pleiomorphic gene clusters common to CVD and OP, we started to analyze the global mRNA and microRNA iliac bone transcriptomes of postmenopausal women. The 1000 transcripts with the strongest negative or positive correlation to aBMD were further analyzed using Ingenuity Pathway Analysis (IPA) (Qiagen, Redwood City, CA, USA) and Database for Annotation, Visualization and Integrated Discovery (DAVID) [21,22]. The genes identified were further characterized based on their known or likely molecular mechanisms in the pathogenesis of CVD, as documented by published data including results from primary human endothelial cells and cardiomyocytes. Finally, we mapped single nucleotide polymorphisms (SNPs) in shared genes associated with both traits, using a genetic pleiotropy-informed conditional False Discovery Rate (condFDR) method supplemented with data from the musculoskeletal knowledge portal (MSK-KP, https://msk.hugeamp.org/, accessed on 11 September 2023).

## 2. Results

### 2.1. Outline of Strategy

**Figure 1:**
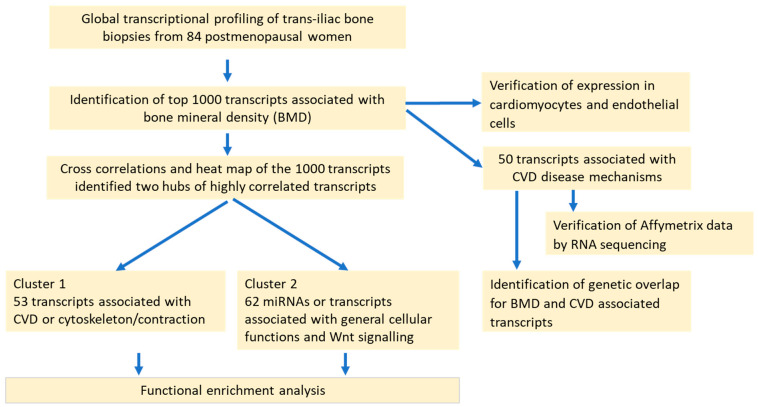
Overview of experimental strategy and work scheme.

The strategy of the study is presented in Figure 1. To analyze the complex interconnection between OP and CVD, we cross-correlated the 1000 BMD transcripts with the strongest correlation to BMD using tools initially developed for the Differential Disease Regulome [23]. One cluster of correlated aBMD-associated transcripts, consisting of mRNAs and microRNAs, was strongly associated with CVD. A second cluster comprised select mRNAs representing, e.g., Wnt signaling pathway modifiers that previously had not or only peripherally been associated with CVD. Relevance of the transcripts with CVD was further evaluated, as indicated in Figure 1.

Using IPA and DAVID, we linked all the 1000 genes in the heat map to specific categories of pathophysiological and biological mechanisms, pathways, and functions with an established or putative role in the pathogenesis of CVD. The analysis revealed that 45 BMD-associated mRNAs and 5 miRNAs were associated with one or several cardiovascular disease mechanisms (Table 1) and categorized within inflammatory response, endothelial function, oxidative stress, dyslipidemia, and calcification. Several of the transcripts with multiple biological and pathophysiological roles therefore appeared in several functional categories, e.g., *PPARA* and *hsa-miR-30c-5p* (Table 1). Further analyses showed that 10 of these transcripts that had strong associations to aBMD and CVD were differentially expressed in healthy individuals and women with established osteoporosis [24], i.e., with at least one fragility fracture (Table 1, in bold).

Some of the genes in Table 1, only recently described as having a role in pathophysiology of CVD, were, e.g., *PPP3CC*-encoding calcineurin, an activator of transcription factor NFATC1. In pulmonary vascular smooth muscle cells, calcineurin/NFaT signaling was reported to regulate the release of Ca^2+^, resulting in increased vascular tone followed by pulmonary arterial hypertension [25]. In comparison, *SPHK1* was involved in sepsis-induced inflammation [26]. Most of the other genes associated with “Inflammatory response” had well-described roles, e.g., *IL15*, encoding a potent pro-inflammatory immune regulatory cytokine exhibiting pro-inflammatory activity [27], and *IKBKG* (*NEMO/IKK-γ*), encoding the inhibitor subunit to IκB kinase (IKK) complex [28]. Other striking features of the genes in Table 1 were their participation in several specific functions related to endothelial cells and the heart. As examples, ATP-binding transporter *ABCA8* is highly expressed in the heart and regulates both cholesterol efflux and high-density lipoprotein cholesterol levels [29] as well as proteins involved in cellular transport, such as IRS1 (insulin receptor substrate 1) [30] and NR3C2 (mineralocorticoid receptor), both central in cellular glucose and Na^+^/K^+^ uptake and exchange [31].

### 2.2. Expression of Shared Genes between Osteoporosis and CVD Analyzed in Primary Endothelial Cells and Cardiomyocytes

**Figure 2:**
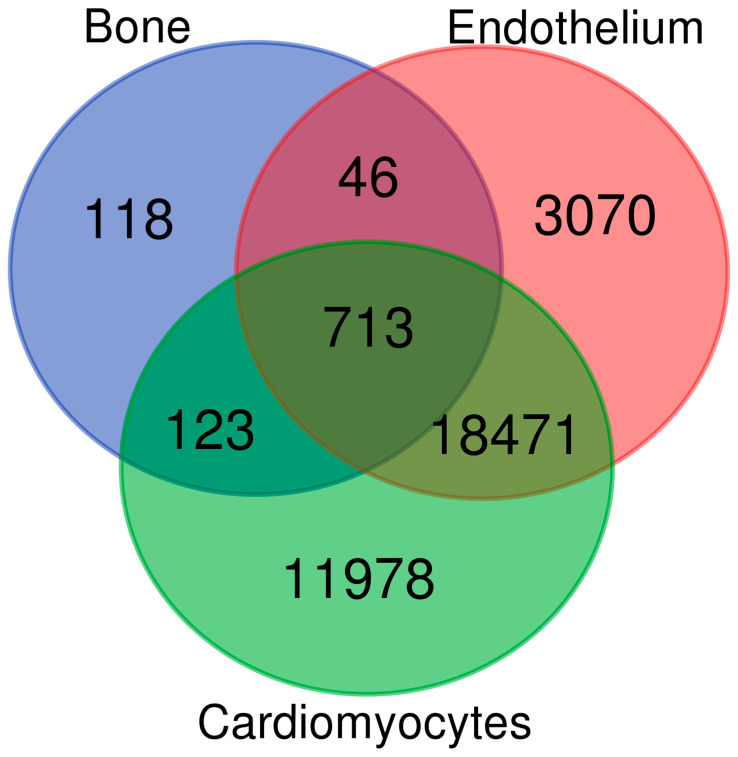
Common transcripts in bone, cardiomyocytes and endothelium. The Venn diagram identifies the number of transcripts among the top 1000 BMD-associated transcripts also expressed in endothelium cells and/or in cardiomyocytes. Of note, 70 out of the 1000 bone transcripts were mature miRNAs not assessed in endothelial cells or in cardiomyocytes.

Endothelial damage and dysfunction are early steps in the development of atherosclerosis leading to reduced cardiac contractility [32]. Also, vascular smooth muscle cells (VSMCs), important for the regulation of cardiac flow, are involved in the early stages of the atherosclerotic process [33]. Since the functionality of cardiomyocytes are completely dependent on an intact microvasculature providing two ways, vital capillary fluxes, the design of their transcriptome ought to be of interest in studying the shared risk genes for CVD. Thus, to further explore the relevance of the discovered genes in CVD, transcript levels in primary microvascular endothelial cells and cardiomyocytes were compared to the bone transcript levels in Table 1 using average expression values adopting the same type of microarray (Table S3). All mRNA genes in Table 1 reached detection levels in the endothelial cells, and all but three were also detected in cardiomyocytes (Table S3). In total, 759 and 836 out of the 1000 BMD-associated genes were also expressed in endothelial cells or cardiomyocytes, respectively (Figure 2). These results suggest a very close similarity between the shared risk genes.

### 2.3. The Identified Genes Common to Postmenopausal Osteoporosis and CVD Are Supported by GWASs

In a recent GWAS [6], using a genetic pleiotropy-informed condFDR method [34], we identified overlap in SNPs from 155 genes that were associated with BMD and cardiovascular disease (CVD)-associated traits and disorders. When we looked for overlap between these genes with the aBMD- and CVD-associated transcripts in the present study, 9 of the 50 genes in Table 1 (star labeled) were detected as detailed in Table S4. Of these, six genes had SNPs within intron or regulatory regions while the remaining three had SNPs in intergenic regions. As a control, we selected three sets of 155 random genes among those correlating to BMD at *p* < 0.05 (out of 4120 genes) and examined how many overlapped with the 50 genes in Table 1. In each set, we found only two common genes, indicating that the nine star-labelled genes were not selected by chance.

### 2.4. Heat Map Identification of the Clusters Representing Closely Correlated Common Genes Present in Osteoporosis and CVD

**Figure 3:**
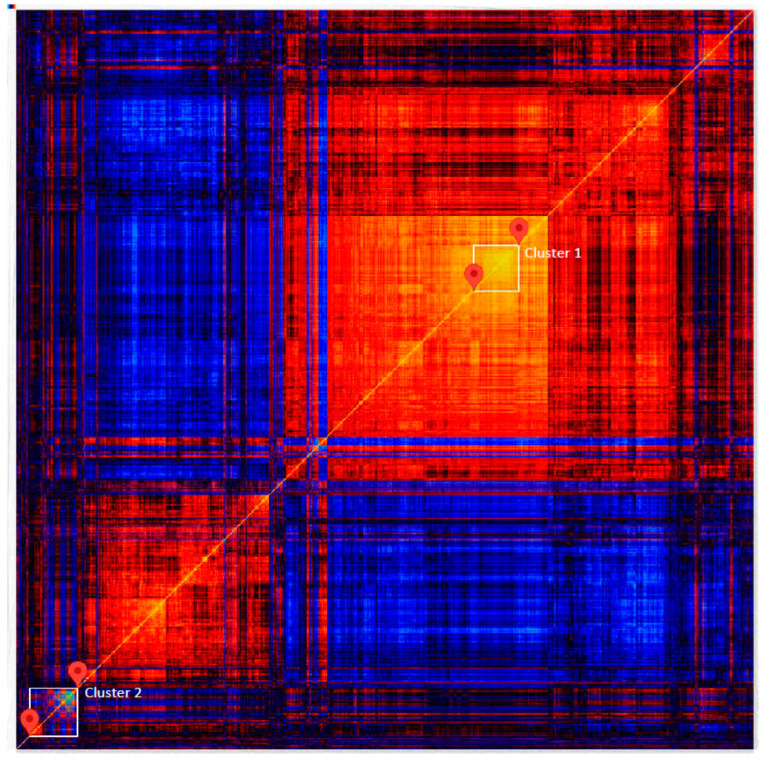
Heat map of closely correlated transcripts. The marked transcript clusters 1 and 2 were selected for functional enrichment analysis. Increasingly positive correlations are from red to yellow and blue shades represent negative correlations, respectively. An interactive version of the heat map is available from the tool menu of the Genomic HyperBrowser (https://hyperbrowser.uio.no/hb/static/hyperbrowser/maps/gautvik_r_most1000_abs/, accessed on 11 September 2023). Tools are provided for creating markers to select either a single pair-wise transcript correlation or a cluster of such correlations. Clicking on a marker creates a pop-up box showing the transcript IDs selected, and the corresponding gene names.

After validation of the applied genomic cluster matrix statistics (Section 4, Figure S1), we studied the 1000 transcripts most significantly correlated to BMD as displayed in the heatmap (Figure 3). The correlated transcripts are presented as red/yellow and bluish, representing positive or negative associations, respectively. Two clusters defining the most significantly correlated transcripts emerged and were subjected to further functional enrichment analysis using IPA and DAVID. These strongest correlated genes represented mRNAs in cluster 1 (53 genes) and mRNAs + microRNAs in cluster 2 (62 genes), as detailed in Tables S1 and S2, respectively. Gene ontology biological process terms (obtained from DAVID) indicated that a major part of cluster 1 genes is involved in pathogenesis related to CVD (Table S1). The results are supported by data from MSK-KP, showing that 20 (38%) of these genes have also SNPs associated with CVD within ±50 kbp of their coding sequence. Twenty-four percent of Table S1 SNPs had the listed BMD or CVD-associated gene as its closest gene.

Taken together, 27 of the 53 unique cluster 1 genes were known to be associated with the gene ontology (GO) process term “cytoskeleton and contraction” in Table S1. Gene symbols marked with “*”, and when present in the same cluster, were all intercorrelated, suggesting interactive functions. As also shown in Table S1, knockout of several of these genes in mice have effects on CVD or the skeleton. DAVID functional enrichment analysis with cluster 1 genes identified the KEGG pathway, adrenergic signaling in cardiomyocytes, as significant (adjusted *p*-value = 0.033).

In cluster 2, the largest number of transcripts was represented by miRNAs, all with a significantly strong association with BMD, but so far with a hitherto unproven direct role in CVD pathogenesis. The microRNAs of cluster 2 appeared together with mRNAs from genes known to be of central importance for bone biology, such as the Wnt signaling pathway (*SOST*, *DKK1*, *WIF1*, *FZD8*) (Table S2). In addition, cluster 2 contained transcripts without prior established association with BMD, osteoporosis or bone cell signaling (e.g., *IL31RA*, *ELTD1*, *ZNF277*). DAVID functional enrichment analysis of cluster 2 mRNAs identified the KEGG pathway and Wnt signaling as significant (adjusted *p*-value = 0.047). We performed enrichment analysis of the cluster 2 miRNAs separately using the miRPath function within DIANA TOOLS [35]. As shown in Table S2, the Wnt signaling pathway again was significant (*p*-value: 2.14 × 10^−4^) along with other matrix-associated pathways, with ECM–receptor interactions being the most significant (*p*-value: 4.47 × 10^−25^).

### 2.5. Verification of Differential Expressions of CVD-Associated Genes by RNAseq and PCR

We used RNA sequencing to validate the Affymetrix results, and both datasets were correlated to morphometric parameters. Table S5 identifies Pearson correlation coefficients between body mass index (BMI)-adjusted Z-scores (BMI- and age-adjusted BMD) with transcript levels obtained by RNAseq and Affymetrix microchip analysis, respectively. In general, associations identified by Affymetrix analysis are very well reproduced by RNA sequencing. RNAseq data also indicate relative transcript levels of the presented genes.

Since genes in these biopsies have previously been analyzed also by real-time RT-TaqMan PCR analysis [19], we made a comparison between the three technologies of gene expression analysis across 80 different samples. As shown in Table S6, Affymetrix signal values correlated better to RNAseq reads than to PCR data. And, interestingly, for the tested genes, more “Affymetrix transcripts” showed stronger correlations with corresponding PCR data than did similar “RNAseq transcripts”.

## 3. Discussion

By analyzing trans-iliacal biopsies of clinically well-characterized postmenopausal women [19], we identified aBMD-associated genes with overlapping functions in the pathogenesis of OP and CVD presented as shared risk genes.

A striking feature of the genes in Table 1 (cluster 1) is their participation in several specific biological actions related to endothelial cells and heart functions. Cluster 1 genes are characterized by a broad specter of biological actions and their clinical relevance for CVD is therefore not always easily appreciable. However, support for the pleiotropic function of cluster 1 genes exists since profiling the protein-encoding transcriptome of human smooth muscle [36] detected 64.7% of the genes. Moreover, among the 53 aBMD-associated genes in cluster 1, 28 were associated with CVD based on their GO biological process term or CVD-related phenotype of associated SNPs (Table S1).

Cluster 2 in the heat map (Figure 3) contained all the microRNAs strongly associated with a select group of genes with often general cellular functionality. They have established roles in bone remodeling and growth comprising canonical Wnt signaling in osteoblasts/osteocytes (*SOST*, *DKK1*, *WIF1*), which are of major importance in bone remodeling and osteoporosis pathophysiology. Interestingly, Wnt signaling is also critical for several pathological events leading to cardiovascular disease, e.g., Wnt Inhibitory Factor 1 (WIF1) [37] (Table 1). Sclerostin (Table S2), another inhibitor of the bone anabolic Wnt signaling pathway, is a target for romosozumab treatment against osteoporosis. A recent large GWAS of osteoporotic patients treated with romosozumab to reduce serum sclerostin levels suggests increased risk for myocardial infarction (OR 1.35 [95% CI 1.01–1.79]) [16].

The RANKL system and its associated genes are present in osteoblasts, but the signaling system is especially characteristic for osteoclasts and macrophages, which constitute a very small population of bone cells, and their contribution is therefore less apparent in the present data [38].

Table 1 summarizes for the first time the mRNAs and miRNAs that are not only strongly correlated to aBMD, but also have much broader systemic actions outside the skeleton, including an established association to the pathogenesis of CVD. For instance, *NR3C2*, which encodes the mineralocorticoid receptor, has a recognized association to CVD [39], but is hitherto not known to affect BMD or be involved in the pathogenesis of OP. Similarly, *PDE5A* (phosphodiesterase-5A) is expressed in smooth muscle cells and plays an important role in the regulation of vascular tone, where inhibition improves endothelial function [40]. Although studies indicate that peak bone mass has stronger heritability than postmenopausal OP [41,42], many of the cluster 1 and 2 genes were identified via MSK-KP to contain genetic variants associated with OP/BMD (Tables S1 and S2). Also, several of the BMD-associated genes were found in the very comprehensive study by Morris et al. [43], such as *CD44*, *FANCC*, *PPARA*, *PPP3CC PRDX2*, *PDK4*, *ABCA8*, *IRS1*, *PPARGC1B*, *DICER1*, *PPP1CB* and *TOB1* (Table 1).

The largest number of detected genes are associated with inflammation, but some of the most prominent, such as *FANNC*, *IL15*, *PPP3CB*, *PP3CC* and *TMG2*, have no established or described role in bone and are thus worthy of further studies. For instance, *PP3CC* positively influences the protein level of *ITPKC*, a susceptibility gene of Kawasaki disease encoding a kinase that negatively regulates intracellular Ca^2+^ levels and inhibits the calcineurin-dependent activation of NFAT by phosphorylating IP3 [44]. The plausible roles of these genes in OP include the demonstrated involvement of NFATc1 in the regulation of bone mass acting on both osteoblasts and osteoclasts [45]. A recent paper studying the transcriptomes in human monocytes, mesenchymal cells or endothelial cells from healthy persons or donors with osteoporosis or CVD also identified immune and inflammatory responses to be the most significant common denominator of disease, thus corroborating results from the current study [46].

Correlation of all the genes in Table 1 to patients with established osteoporosis and fragility fractures shows that 10 of them are differentially expressed and may represent core risk genes for both clinical OP and CVD. However, their pathophysiological molecular actions in bone and vasculature are not well known. Thus, the two diseases should not be considered as clinically distinct.

Several studies have indicated the involvement of Transglutaminase TGM2 (Table 1) during the initial phase of wound healing and inflammation [47]. *SPHK1* (sphingosine kinase 1) (Table 1) encodes a protein that catalyzes the phosphorylation of sphingosine to form sphingosine-1-phosphate (S1P), which mediates the down-regulation of JNK activity, and serves to dampen inflammation and tissue injury [26]. Both appear also to be involved in CVD. *TGM2* and *SPHK1* have, e.g., both been associated with hypertension [48,49]. A recent meta-analysis [50] comprising 1.3 million individuals has identified rare variants affecting blood pressure with effects ~8 times larger than common variants, and several of their associated genes are present among the 1000 genes that we analyzed in depth. One group is represented by missense variants (*NR3C2* (*GR*), *DOCK4*, *SOS2*) with effect sizes > |0.2|. Other variants, also present among the 1000 genes, have normal amino acid sequences and are located near or in genes such as *KCNH2*, *SLC25A37*, *PPTC7* and *SLC4A1*. In Table S3, we document that all the pleiotropic mRNAs of Table 1 are expressed in cultured, human primary endothelial cells from three donors and all, but three are also present in cardiomyocytes.

### 3.1. Evaluation of Bone Tissue Composition and Heterogeneity

The trans-iliacal biopsies contain outer walls of cortical bone connected with trabeculae and harbor-interspersed bone marrow cells, nerves and blood circulation. Estimation of the osteocyte fraction in healthy and osteoporotic women shows similar results [19], as does the ratio of bone marrow and blood cells (about 20%) (RNA sequence data). Osteoclast-specific transcripts (e.g., *CALCR* and *RANKL* (*TNSF11*)) are not detected due to the relatively low representation of osteoclasts in bone. Furthermore, we have previously shown by histocytometry that the numbers of osteocytes are not distinct between osteoporotic and healthy individuals, and that levels of several osteocyte-specific transcripts are similar in bone from osteoporotic and healthy women [19]. Therefore, we conclude that the bone cell composition is not distinct between healthy and patients and cannot explain the difference in transcript profiles.

### 3.2. Strength and Weaknesses of the Study

We present the most extensive study of postmenopausal women subjected to bone biopsies and transcriptome analysis using Affymetrix microchips supported by RNA sequencing, and the results show a high degree of concordance. The reliability of the results is further assessed by comparing PCR to the microchip and RNAseq data. However, the number of participants may still be considered limited. Another limitation is that the study does not comprise both sexes, and males are still more susceptible to CVD than females, even if the difference in prevalence is diminishing. None of the detected susceptibility genes are, however, apparently linked to sex. Therefore, the results should also have bearing on the male population above 50 years of age and especially for the ca. 20% who develop primary OP. Admittedly, bone biopsies contain a heterogeneous cell population, mostly representing cells of the hematological and immunological lineages, in addition to bone cells. However, after assessing their contribution, we conclude that they would not substantially impact the results. Possible immunological aspects, including inflammation, have been implied both in CVD conditions and OP [51]. Two groups of transcripts representing “Inflammation”/“Oxidative stress response” are presented in Table 1, and available, although limited, detailed information is given above. The most significant confounding factors related to BMD are age and BMI. However, since the Pearson correlation coefficient between lumbar spine (L2–L4) T-score and age and BMI-adjusted T-score is strong (r = 0.92), we consider the effect as negligible. As lifestyle factors are found to be very similar between groups of bone donors as previously thoroughly documented [19], and the study by far is the hitherto largest published group representing unrelated, ethnic Norwegian women, the work is considered robust.

In recent years, it also has become evident that the gut microbiome may affect bone metabolism [52,53]. Although the participants reported intakes of traditional Norwegian food, it cannot be ruled out entirely that diet variations may affect gut microbiomes of bone donors, possibly impacting the outcome of the study.

Bone biopsies are representative of trabecular bone such as vertebra, the most common site disposed for the osteoporotic process and early fragility fracture. However, the pelvis is not as weight-bearing as, e.g., the spine, and thus possible confounding factors of exercise-induced changes in gene expression are reduced or not present. Our findings in humans are supported by results from GWASs [6,54], and they are consistent with previous results of shared genes between aBMD, CVD and diabetes [6].

## 4. Materials and Methods

### 4.1. Bone Donors and Biopsies

The unrelated Norwegian Caucasian women (50–86 years), representing a cohort with varying bone mineral densities (BMDs) and free of primary diseases known to affect the skeleton, were consecutively recruited at the Lovisenberg Diaconal Hospital, the Out-patient Clinic, Oslo. A detailed questionnaire, filled out during the consultation, contained information regarding nutrition and additional dietary intake (e.g., vitamins and minerals), physical activity and lifestyle risk factors (alcohol and smoking). As previously described [24], 39 of the women were osteoporotic (T-score ≤ −2.5), 27 healthy (T-score > −1), and 18 were classified as an intermediate, osteopenic group. Detailed clinical and laboratory parameters were previously published for each group [24]. No participants reported cardiac or pulmonary symptoms. Several of the healthy and osteoporotic women were also included in a project to study the impact of 12 weeks of heavy-load exercise on muscle strength, balance performance and BMD. Both groups reached the same results regarding all test parameters [55,56]. Also, the lifestyle parameters were similar between groups [24]. Strict inclusion criteria resulted in the rejection of two-thirds of 301 examined women due to medication or diseases other than primary osteoporosis [19]. Thus, 84 trans-iliacal bone biopsies were obtained ad modum Bordier [19,57] from os ileum at a predefined location (2.0 cm from crista iliaca and 2 cm from spina iliaca). All bone biopsies were performed in the morning on fasting individuals. The biopsies were cylindrical, 0.8 cm in diameter with an average length of about 1.5 cm. Connective tissue and muscle were removed before being frozen in liquid nitrogen. Analyses from ileum biopsies of right and left side from the same person showed more than 98% identical global transcription profiles [19]; the results were compiled.

### 4.2. Data from Primary Endothelial Cells and Cardiomyocytes

For primary endothelial cells expression data, we took advantage of repository data of Human Dermal Microvascular Endothelial Cells obtained from Lonza (Basel, Switzerland) as described [58]. We obtained primary data in the form of Affymetrix CEL files from the ArrayExpress repository (GSM700326, GSM734106, and GSM734107). Robust microarray analysis (RMA) yielding normalized log2 transformed signal intensities was applied for the normalization of data.

Expression data from cultured iCell cardiomyocytes derived from WA09 human embryonic stem cells were obtained from geo_accession GSE33325 (GSM824086, GSM824087, GSM824088, GSM824089). Genes with average log2 signal value < 5 were excluded. The same array type and normalization procedure were used for both endothelial cells and cardiomyocytes.

### 4.3. RNA Purification and Gene Expression Analysis of Bone Biopsies

Total RNA was purified and subjected to analysis on HG-U133 plus 2.0 microarrays (Affymetrix, Santa Clara, CA, USA), as previously described [19]. In brief, bone biopsies cooled with liquid N2 were pulverized in a mortar and homogenized in Trizol reagent (Life Technologies, Gaithersburg, MD, USA), as previously described [19]. RNA was extracted according to the manufacturer’s instructions with a yield of 80–250 μg total RNA. RNA was further purified using the miRNeasy micro kit (Qiagen, Hilden, Germany) to remove organic components. The RNA integrity was checked using Agilent 2100 BioAnalyzer (Agilent Technologies, Waldbronn, Germany) and the RNA Nano LabChip assay. All samples had an RNA integrity number (RIN) of about 7 or higher.

The data for postmenopausal biopsies were submitted to the European Bioinformatics Institute (EMBL-EBI) ArrayExpress repository, ID: E-MEXP-1618.

The samples subjected to Affymetrix analysis were recently analyzed also by RNA-sequencing, and these data were used to confirm the microarray results.

RNA sequencing was carried out by paired-end sequencing. Read counts per known transcript were determined and pasted into a matrix; in total, there were 57,820 transcripts. This matrix was then FPKM-transformed by correcting for total read counts per sample and transcript length using the following formula: (10^9^ ∗ Counts)/(total reads ∗ length of transcript). All transcripts with a FPKM ≥ 1 in at least 10 samples were retained; thus, 38,817 transcripts were discarded.

Mature miRNAs were analyzed as previously described [59], employing a PCR-based method with TaqMan miRNA LDA Arrays A and B following the manufacturer’s instructions (Applied Biosystems, Foster City, CA, USA).

### 4.4. Calculations, Statistics and the Web Application

Pearson correlation coefficients (r) were computed between log2 transformed signal values of all genes (~23,000 probe sets) and BMI-adjusted total hip BMD Z-score (age-adjusted BMD) in 84 postmenopausal women. The BMI-adjusted Z-scores were computed by first regressing the Z-score and BMI (with intercept), and then defining the residuals as the BMI-adjusted Z-scores. The 1000 most significantly correlated probe sets were subjected to bioinformatics analysis to identify transcripts associated with specific pathophysiological and biological mechanisms, pathways, and functions, having an established or putative role in the pathogenesis of CVD employing DAVID and IPA.

The log2 transformed signal values of the selected 1000 probe sets were further inter-correlated as Pearson correlation coefficients r. Rows and columns of the resulting matrix of pair-wise correlation coefficients were clustered hierarchically using average linkage and 1 − |r| as distance measure. Transcripts were thus clustered together based on correlation values, regardless of the direction of correlation as visualized in the heat map (Figure 3). Median and mean correlation values against BMI-adjusted Z-scores were both |0.30|, while the minimum and maximum absolute correlation values were |0.27| and |0.51|, respectively.

### 4.5. Validation of Genomic Cluster Matrix Statistics

When correlating BMD with a large number of randomly selected transcripts that are not chosen by their correlation to BMD, a plot of the r-values on the *x*-axis against the number of specific r-values would be expected to show a single peak centered around r = 0. In contrast, when selecting the set of transcripts that most significantly associates with BMD, two separate peaks are to be expected with negative or positive r-values, if there was a biological association. When this analysis was performed on our dataset using first a cluster matrix made from the 1000 transcripts with the lowest correlation to BMD, a single peak was indeed observed centered around r = zero when plotting Pearson correlation coefficients against the number of correlations (Figure S1A). The plot consisting of 1000 genes with the highest correlation to BMD yielded 2 peaks, each appearing asymmetrical with maxima clearly off the r = 0 value, suggesting non-random results with distributions as positive (red) or negative (blue) correlations (Figure S1B).

### 4.6. Identification of Genetic Overlap for BMD- and CVD-Associated Transcripts

As an alternative strategy to identify genes associated with both BMD and CVD, we used a conditional False Discovery Rate (FDR) approach [34]. This method relies on summary statistics and identifies the enrichment of SNPs associated with BMD as a function of association with CVD and vice versa. The method leverages genome-wide association study (GWAS) results from related phenotypes without the need for larger datasets with a corresponding reduction in FDR [6]. For the current study, we selected SNPs associated with both CVD and BMD at an informed conditional FDR of 10%.

## 5. Conclusions

The present identification of overlapping genetic factors presented as mRNAs and microRNAs, underlying CVD and OP, are supported by GWASs and protein profiling, and will help explain the frequent comorbidity and possibly increase the awareness of potential drug side effects. The present results may furthermore encourage continued work to discover improved, and possibly common, therapy of both chronic major disorders.

## Figures and Tables

**Table 1:** Female bone mRNAs and miRNAs strongly associated with aBMD, with an established role in cardiovascular disease pathophysiologic processes.

Inflammatory Response			Oxidative Stress Response			Dyslipidaemia			Fibrosis			Osteoclastogenesis and Calcification			Endothelial Function		
	r	*p*		r	*p*		r	*p*		r	*p*		r	*p*		r	*p*
*CD44* *	0.28	9.3 × 10^−3^	*ETFDH*	−0.29	7.5 × 10^−3^	***ACSL3***	0.45	1.6 × 10^−5^	***ACADM***	−0.30	6.3 × 10^−3^	*ACVR2A*	−0.29	7.7 × 10^−3^	*NR3C2* *	−0.30	6.3 × 10^−3^
*FANCC* *	0.33	2.2 × 10^−3^	*MAOB*	−0.32	2.8 × 10^−3^	***PDK4***	−0.34	1.7 × 10^−3^	*AR*	−0.27	1.3 × 10^−2^	*GATA1*	0.30	6.2 × 10^−3^	*PDE5A*	−0.31	4.2 × 10^−3^
*IKBKG*	0.29	7.1 × 10^−3^	*OGG1*	−0.27	1.3 × 10^−2^	***HADHB***	−0.32	3.1 × 10^−3^	*CREM*	−0.31	4.2 × 10^−3^	*HIVEP2* *	−0.28	9.8 × 10^−3^	*PPP3CC*	−0.33	2.4 × 10^−3^
***IL15***	−0.29	6.8 × 10^−3^	*PARK7*	−0.27	1.4 × 10^−2^	*ABCA8* *	−0.29	7.2 × 10^−3^	*DICER1*	−0.30	5.9 × 10^−3^	***TOB1***	−0.28	9.3 × 10^−3^			
*ITGAV*	−0.28	9.6 × 10^−3^	*PRDX2*	0.37	5.0 × 10^−4^	***ACADM***	−0.30	6.3 × 10^−3^	*NPY1R*	−0.28	1.1 × 10^−2^	*WIF1*	0.37	5.0 × 10^−4^			
*PDE4D* *	−0.28	1.1 × 10^−2^	*PRDX6*	−0.34	1.8 × 10^−3^	*CA1*	0.27	1.4 × 10^−2^	*NR3C2* *	0.28	2.0 × 10^−3^	***WWP1***	−0.37	6.0 × 10^−4^			
*PDE5A*	−0.31	4.2 × 10^−3^	*RRM2B*	−0.30	1.1 × 10^−2^	*IRS1*	−0.34	1.6 × 10^−3^	***PPARA***	−0.30	6.3 × 10^−3^						
*PDE7A*	−0.29	7.1 × 10^−3^	*SNCA*	0.34	1.6 × 10^−3^	***PPARA***	−0.34	1.9 × 10^−4^	*PPP1CB* *	−0.30	5.7 × 10^−3^						
***PPARA***	−0.34	1.7 × 10^−3^	*SRXN1*	0.29	7.9 × 10^−3^	*PPARGC1B*	−0.35	9.0 × 10^−4^	*RRM2B*	−0.30	1.1 × 10^−2^						
***PPP3CB***	−0.39	3.0 × 10^−4^				*PTPN1* *	0.35	1.3 × 10^−3^	*SMAD2*	−0.29	6.5 × 10^−3^						
*PPP3CC*	−0.33	2.4 × 10^−3^							***TRDN***	−0.28	1.1 × 10^−2^						
*PTPN11* *	−0.32	3.3 × 10^−3^							*miR-30c-5p*	−0.38	2.1 × 10^−5^						
*SPHK1*	0.28	1.1 × 10^−2^															
*TGM2*	0.31	3.7 × 10^−3^															
*miR-30c-5p*	−0.38	1.4 × 10^−4^															
*miR-365a-3p*	−0.37	1.5 × 10^−4^															
*miR-484*	−0.41	2.2 × 10^−5^															
*miR-494*	0.47	8.7 × 10^−7^															
*miR-92a-3p*	−0.39	4.8 × 10^−5^															

* Transcripts that correlate with aBMD and have SNPs associated with both BMD and a CVD-related trait. Bold indicates significantly different expression in healthy and osteoporotic women with at least one fragility fracture (i.e., clinically established osteoporosis). R and *p* values (nominal) refer to correlation with BMI-adjusted total hip Z-score.

## Data Availability

The bone transcriptome dataset analyzed by microarrays are available in the ArrayExpress repository (EMBL-EBI; ID: E-MEXP-1618), https://www.ebi.ac.uk/biostudies/arrayexpress/studies/E-MEXP-1618, accessed on 22 November 2022. The bone RNA-Seq data are publicly available at https://www.ncbi.nlm.nih.gov/bioproject/, (SRA accession number: PRJNA764663) accessed on 22 November 2022.

## Supplementary Materials

The supporting information can be downloaded at: https://www.mdpi.com/article/10.3390/ijms25105554/s1.
