# Supplementary material for: Identification of Transcripts with Shared Roles in the Pathogenesis of Postmenopausal Osteoporosis and Cardiovascular Disease
Source: Int J Mol Sci. 2024 May 20;25(10):5554. doi: 10.3390/ijms25105554 (PMC11121938; doi:10.3390/ijms25105554)
Supplement: Supplementary file 1 [file ijms-25-05554-s001.zip › ijms-2984048-supplementary.pdf]

SUPPLEMENTARY MATERIAL

Identification of Transcripts with Shared Roles in the Pathogenesis of Postmenopausal Osteoporosis and Cardio-vascular Disease

Sjur Reppe, Sveinung Gundersen, Geir Kjetil Sandve, Yunpeng Wang, Ole A. Andreassen, C. Medina-Gomez, F. Rivadeneira, Tor P. Utheim, Eivind Hovig, Kaare M. Gautvik

Table S1. Cluster 1 genes

Probe Set ID	r	Gene Symbol	Gene Title	GO biological process term	CVD associated SNP	CVD related Phenotype	eBMD associated SNP
235183_at	-0.28	FILIP1	filamin A interacting protein 1		6:76012239:T:A	Diastolic blood pressure	
236856_x_at	-0.32	---	---				
218168_s_at	-0.31	*ADCK3 (COQ8A)**	aarF domain containing kinase 3	cell death /// phosphorylation /// high expression in heart mediate p53 induced apoptosis	rs2813960	Diastolic blood pressure	rs2855562
225450_at	-0.32	*AMOTL1*	angiomotin like 1	establishment of cell polarity involved in ameboidal cell migration /// hippo signaling cascade /// positive regulation of blood vessel endothelial cell migration ///conduct disorder ADHD			
202920_at	-0.28	*ANK2**	ankyrin 2, neuronal	regulation of heart rate /// atrial septum development /// cellular calcium ion homeostasis /// signal transduction /// axon guidance /// protein localization /// positive regulation of gene expression /// regulation of cardiac muscle contraction by regulation of the release of sequestered calcium ion /// cardiac arythmia			
203296_s_at	-0.28	*ATP1A2**	ATPase, Na+/K+ transporting, alpha 2 polypeptide	neurotransmitter uptake /// regulation of the force of heart contraction /// regulation of respiratory gaseous exchange by neurological system process /// ATP biosynthetic process /// cation transport /// potassium ion transport /// sodium ion transport /// regulation of muscle contraction			
201848_s_at, 201849_at	-0.29 -0.35	*BNIP3*	BCL2/adenovirus E1B 19kDa interacting protein 3	response to hypoxia /// apoptotic DNA fragmentation /// chromatin remodeling /// anti-apoptosis /// induction of apoptosis by intracellular signals /// negative regulation of survival gene product	rs7912283	Cystolic blood pressure	
228783_at	-0.28	*BVES**	blood vessel epicardial substance	positive regulation of receptor recycling /// cell adhesion /// multicellular organismal development /// muscle organ development /// regulation of cell shape ///vesicle-mediated transport /// regulation of Rac GTPase activity ///heart development muscle specific	rs78139700	Creatine kinase	rs364663
225105_at	-0.30	*C12orf75*	chromosome 12 open reading frame 75	-			
213547_at	-0.28	*CAND2*	cullin-associated and neddylation-dissociated 2 (putative)	positive regulation of transcription, DNA-dependent /// assembly of ubiquitin ligase complexes	rs6810325	Creatine kinase	rs364663
224352_s_at, 233496_s_at	-0.27 -0.30	*CFL2**	cofilin 2 (muscle)	actin depolymerizing factor			rs10145299
225747_at	-0.27	*COQ10A*	coenzyme Q10 homolog A (S. cerevisiae)		rs56245751	Coronary artery disease	
218970_s_at	-0.31	*CUTC*	cutC copper transporter homolog (E. coli)	copper ion transport /// protein tetramerization /// copper ion homeostasis			
215016_x_at	-0.27	*DST**	dystonin /// dystonin-like	microtubule cytoskeleton organization /// transport /// cell cycle arrest /// cell adhesion /// integrin-mediated signaling pathway /// axonogenesis /// retrograde axon cargo transport /// response to wounding /// maintenance of cell polarity /// regulation of microtubule polymerization or depolymerization /// cytoskeletal linker protein			
219963_at	-0.31	*DUSP13*	dual specificity phosphatase 13	protein dephosphorylation meiosis /// spermatogenesis /// peptidyl-tyrosine dephosphorylation			
232252_at	-0.29	*DUSP27*	dual specificity phosphatase 27(putative)	protein dephosphorylation			
207876_s_at	-0.30	*FLNC**	filamin C, gamma	cell junction assembly /// myofibrillar association	rs3807309	Left ventricular ejection fraction	
213793_s_at	-0.29	*HOMER1**	homer homolog 1 (Drosophila)	skeletal muscle contraction /// phospholipase C-activating G-protein coupled glutamate receptor signaling pathway /// synaptic transmission /// positive regulation of signal transduction /// positive regulation of calcium ion transport via store-operated calcium channel activity /// skeletal muscle fiber development /// response to calcium ion	rs13182512	Type 2 diabetes	
211538_s_at	-0.29	*HSPA2*	heat shock 70kDa protein 2	response to stress /// response to unfolded protein /// male meiosis /// spermatid development /// neuropathy chaperone function			rs3783725
221667_s_at	-0.28	*HSPB8*	heat shock 22kDa protein 8	response to stress /// cell death /// neuropathy chaperone function			
227401_at	-0.28	*IL17D*	interleukin 17D	inflammatory response /// functional in endothelial cells			
225878_at, 226968_at	-0.32 -0.38	*KIF1B**	kinesin family member 1B	ATP catabolic process /// apoptotic process /// microtubule-based movement /// neuron-neuron synaptic transmission /// neuromuscular synaptic transmission /// anterograde axon cargo transport /// defect causes Charcot Marie Tooth neuropathy / highly expressed in heart			
213371_at	-0.28	*LDB3**	LIM domain binding 3	cardio myopathy			rs17335168
218574_s_at	-0.27	*LMCD1**	LIM and cysteine-rich domains 1	negative regulation of transcription from RNA polymerase II promoter /// regulation of cardiac muscle hypertrophy /// positive regulation of calcineurin-NFAT signaling cascade /// cardio myopathy			
243346_at	-0.27	*LMOD3**	leiomodin 3 (fetal)	troponin moudulation			
236523_at	-0.28	*C4orf54(LOC 285556)*	uncharacterized LOC285556				
1552309_a_at, 226103_at	-0.30 -0.32	*NEXN**	nexilin (F actin binding protein)	regulation of cell migration /// regulation of cytoskeleton organization /// function In the actin cytoskeleton	rs34517439	Diastolic blood pressure	
205872_x_at	-0.27	*PDE4DIP*	phosphodiesterase 4D interacting protein	cellular protein complex assembly /// heart development			
210170_at	-0.34	*PDLIM3**	PDZ and LIM domain 3	actin filament organization /// heart development /// cardio myopathy			
213684_s_at	-0.28	*PDLIM5**	PDZ and LIM domain 5	regulation of synapse assembly /// regulation of dendritic spine morphogenesis /// cardio myopathy / contractile element	rs2452600	Coronary artery disease	

Table S1. continued

Probe Set ID	r	Gene Symbol	Gene Title	GO biological process term	CVD associated SNP	CVD related Phenotype	eBMD associated SNP
222572_at	-0.30	*PDP1*	pyruvate dehyrogenase phosphatase catalytic subunit 1	pyruvate metabolic process /// protein dephosphorylation /// regulation of acetyl-CoA biosynthetic process from pyruvate /// peptidyl-threonine dephosphorylation /// small molecule metabolic process			
212915_at	-0.32	*PDZRN3**	PDZ domain containing ring finger 3	neuromuscular junction development /// protein ubiquitination /// protein ubiquitination /// contractile element			
201968_s_at	-0.27	*PGM1*	phosphoglucomutase 1	glycogen biosynthetic process /// glycogen catabolic process /// trehalose biosynthetic process /// glucose metabolic process /// galactose catabolic process /// small molecule metabolic process /// activates pyrophosphatase			
203335_at	-0.34	*PHYH*	phytanoyl-CoA 2-hydroxylase	fatty acid alpha-oxidation /// cellular lipid metabolic process /// small molecule metabolic process /// oxidation-reduction process			
204612_at	-0.27	*PKIA*	protein kinase (cAMP-dependent, catalytic) inhibitor alpha	negative regulation of transcription from RNA polymerase II promoter /// negative regulation of protein kinase activity /// regulation of G2/M transition of mitotic cell cycle /// phosphorylation /// negative regulation of protein import into nucleus /// negative regulation of catalytic activity /// Belong to PKI family			
205478_at	-0.28	*PPP1R1A*	protein phosphatase 1, regulatory (inhibitor) subunit 1A	carbohydrate metabolic process /// glycogen metabolic process /// signal transduction			
209633_at, 209632_at	-0.28<br>-0.31	*PPP2R3A*	protein phosphatase 2, regulatory subunit B", alpha	protein dephosphorylation	rs687339	Coronary artery disease	
200732_s_at	-0.34	*PTP4A1*	protein tyrosine phosphatase type IVA, member 1	cell cycle /// multicellular organismal development /// positive regulation of cell migration /// peptidyl-tyrosine dephosphorylation			
204217_s_at,<br>34408_at	-0.27<br>-0.30	*RTN2*	reticulon 2	signal transduction /// cell death /// Deliver EAAC1 from ER to cell surface	rs344790	Left ventricular end-diastolic volume (BSA-indexed)	rs12373539
206306_at	-0.27	*RYR3**	ryanodine receptor 3	cellular calcium ion homeostasis /// striated muscle contraction /// protein homotetramerization /// transmembrane transport ///calcium ion transmembrane transport /// cellular response to calcium ion /// cellular response to magnesium ion /// cellular response to caffeine /// cellular response to ATP			
204979_s_at	-0.27	*SH3BGR*	SH3 domain binding glutamic acid-rich protein	protein complex assembly /// cardiac skeletal muscle	rs4818025	LDL cholesterol	
202565_s_at	-0.31	*SVIL**	supervillin	cytoskeleton organization /// skeletal muscle tissue development /// myogenesis			
212730_at	-0.27	*SYNM**	synemin, intermediate filament protein	intermediate filament cytoskeleton organization /// localized to vessels and muscle cells			
225895_at	-0.34	*SYNPO2**	synaptopodin 2	sarcoplasmic reticulum function	rs9799664	Diastolic blood pressure	
230611_at	-0.30	*SYPL2**	synaptophysin-like 2	transport /// cellular calcium ion homeostasis /// sarcoplasmic reticulum function	rs4970837	LDL cholesterol	
202289_s_at	-0.30	*TACC2**	transforming, acidic coiled-coil containing protein 2	microtubule cytoskeleton organization /// cell proliferation /// cerebral cortex development /// neurogenesis /// interkinetic nuclear migration /// astral microtubule organization /// regulation of microtubule-based process	rs11598094	Coronary artery disease	rs4077606
226388_at	-0.28	*TCEA3*	transcription elongation factor A (SII), 3	regulation of transcription from RNA polymerase II promoter /// translational elongation /// regulation of transcription elongation, DNA-dependent /// cofactor for RNA polym II	rs61778885	LDL cholesterol	
238867_at	-0.33	*TMEM182*	transmembrane protein 182		2:103329813:T:C	Heart rate	
238079_at	-0.28	*TPM3**	tropomyosin 3	cellular component movement /// regulation of muscle contraction /// muscle filament sliding	rs61812598	Plasma C-reactive protein	
222919_at	-0.28	*TRDN**	triadin	muscle contraction /// negative regulation of ryanodine-sensitive calcium-release channel activity /// Ca uptake in heart			
236972_at	-0.28	*TRIM63**	tripartite motif containing 63, E3 ubiquitin protein ligase	signal transduction /// protein ubiquitination /// associates to muscle contractile elements			rs7546500
205356_at, 226902_at	-0.30	*USP13*	ubiquitin specific peptidase 13 (isopeptidase T-3)	regulation of transcription, DNA-dependent /// proteolysis /// ubiquitin-dependent protein catabolic process /// cell proliferation /// regulation of autophagy /// melanocyte differentiation /// protein stabilization /// protein K63-linked deubiquitination /// intracellular peptidase			
225665_at,<br>225662_at	-0.28<br>-0.31	*ZAK**	sterile alpha motif and leucine zipper containing kinase AZK	cell cycle checkpoint /// DNA damage checkpoint /// MAPK cascade /// activation of MAPKK activity /// protein phosphorylation /// response to stress /// cytoskeleton organization /// cell cycle arrest /// actin fiber organization	rs2358084	Diastolic blood pressure	

These genes in Cluster 1 are defined by the columns 618-679 and rows 319-380 within the matrix. The second column displays Pearson correlation coefficients (r) between indicated transcripts and total hip BMI adjusted Z-score. DAVID Gene Functional Classification was used to identify the gene ontology (GO) biological process term of the genes. *Genes related to cytoskeleton / contraction. **SNP also related to estimated BMD (eBMD) at genome-wide significance (GWS, $p<5E-8$)

Presence of SNPs related to CVD in each gene was obtained from the Musculoskeletal Knowledge portal: https://msk.hugeamp.org/ In cases with several affected CVD associated phenotypes only the most significant (at GWS) is presented. The region tested for associated SNPs includes the coding sequence of the gene plus 50kb of upstream and downstream flanking sequences.

Table S2. Cluster 2 genes

Probe Set ID	r	Gene Symbol	Gene Title	GO biological process term
207172_s_at,<br>207173_x_at	0.31<br>0.33	*CDH11*	cadherin 11, type 2, OB-cadherin (osteoblast)	
204320_at	0.27	*COL11A1*	collagen, type XI, alpha 1	
204602_at	0.44	*DKK1*	dickkopf 1 homolog (Xenopus laevis)	
1556820_a_at,<br>1556821_x_at,<br>1569600_at	-0.31<br>-0.32<br>-0.34	*DLEU2*	deleted in lymphocytic leukemia 2 (non-protein coding)	
219134_at	-0.34	*ELTD1*	EGF, latrophilin and seven transmembrane domain containing 1	signal transduction /// cell surface receptor signaling pathway /// G-protein coupled receptor signaling pathway /// neuropeptide signaling pathway
226170_at	0.30	*EYA3*	eyes absent homolog 3 (Drosophila)	
204379_s_at	0.28	*FGFR3*	fibroblast growth factor receptor 3	
227405_s_at	0.30	*FZD8*	frizzled family receptor 8	
243541_at	-0.32	*IL31RA*	interleukin 31 receptor A	
221150_at	0.37	*MEPE*	matrix extracellular phosphoglycoprotein	skeletal system development /// negative regulation of bone mineralization /// biomineral tissue development
205959_at	0.27	*MMP13*	matrix metallopeptidase 13 (collagenase 3)	
37408_at	0.28	*MRC2*	mannose receptor, C type 2	Endocytosis
227394_at	0.27	*NCAM1*	neural cell adhesion molecule 1	
219747_at	0.27	*NDNF*	neuron-derived neurotrophic factor	
223869_at	0.43	*SOST*	sclerostin	
201645_at	0.30	*TNC*	tenascin C	
202365_at	0.27	*UNC119B*	unc-119 homolog B (C. elegans)	transport /// protein transport /// cell projection organization /// lipoprotein transport /// cilium morphogenesis
204712_at	0.37	*WIF1*	WNT inhibitory factor 1	regulation of transcription, DNA-dependent /// multicellular organismal development /// Wnt receptor signaling pathway /// positive regulation of fat cell differentiation
215887_at	0.33	*ZNF277*	zinc finger protein 277	regulation of transcription, DNA-dependent
228528_at	-0.35			
230127_at	-0.35			
230319_at	0.31			
230503_at	-0.35			
230640_at	0.30			
235538_at	0.27			
AFFX-M27830_5_at<br>AFFX-M27830_M_at	0.37<br>0.44		28S ribosomal RNA	
228464_at	-0.32	*MIR3685*	hsa-mir-3685	
rnu24	0.28			
rnu44	-0.42			
rnu48	-0.40			
hsa-mir-15b-5p	-0.39			
hsa-mir-181a-5p	0.41			
hsa-mir-218-5p	0.34			
hsa-mir-22-3p	0.28			
hsa-mir-27a-5p	-0.39			
hsa-mir-28-3p	-0.36			
hsa-mir-29a-3p	0.39			
hsa-mir-29c-3p	0.37			
hsa-mir-29c-5p	0.37			
hsa-mir-30a-5p	-0.35			
hsa-mir-30b-5p	-0.33			
hsa-mir-30c-5p	-0.35			
hsa-mir-324-3p	0.44			
hsa-mir-328	-0.43			
hsa-mir-331-3p	-0.47			
hsa-mir-335-3p	-0.32			
hsa-mir-340-3p	-0.36			
hsa-mir-342-3p	-0.39			
hsa-mir-365a-3p	-0.34			
hsa-mir-425-3p	-0.40			
hsa-mir-451a	0.36			
hsa-mir-484	-0.37			
hsa-mir-494	0.43			
hsa-mir-625-3p	-0.40			
hsa-mir-627	0.41			
hsa-mir-652-3p	0.39			
hsa-mir-92a-3p	-0.36			
hsa-mir-939	0.34			
hsa-mir-99b-5p	-0.30			
hsa-mir-10a-5p	-0.27			
hsa-mir-139-5p	-0.30			
hsa-mir-15a-5p	0.51			

Potentially altered KEGG molecular pathways by the displayed miRNAs as identified by Diana miRPath.

#	KEGG pathway	p-value	#genes	#miRNAs
1.	ECM-receptor interaction (hsa04512)	4.47e-25	47	23
2.	Glycosaminoglycan biosynthesis - heparan sulfate (hsa0053413)	1.51e-16	13	18
3.	Mucin type O-Glycan biosynthesis (hsa00512)	3.83e-07	16	17
4.	Focal adhesion (hsa04510)	3.83e-07	113	30
5.	Glioma (hsa05214)	2.35e-04	39	25
6.	Wnt signaling pathway (hsa04310)	2.14e-04	79	26
7.	Pathways in cancer (hsa05200)	3.11e-04	156	31
8.	N-Glycan biosynthesis (hsa00510)	4.03e-04	25	20
9.	ErbB signaling pathway (hsa04012)	5.12e-04	48	28
10.	Glycosaminoglycan biosynthesis - keratan sulfate (hsa00533)	5.39e-04	10	10

Kegg pathways affected Parentheses denote KEGG pathway number

The Cluster 2 genes are defined by columns 19-84 and rows 914-979 within the matrix. The second column displays Pearson correlation coefficients (r) between indicated transcripts and total hip BMI adjusted Z-score. DAVID Gene Functional Classification was used to identify the Gene Ontology (GO) biological process term of the genes. The DIANA miRPath software was used to identify potentially altered molecular pathways by the listed microRNAs target genes (shaded insert panel). The results are based on the number of targeted genes involved in a determined pathway and compar that number with the expected number of genes, depending on how many genes constitute the specific Pathway. A chi-square test returns a distinct p-value for each pathway.

Table S3. Comparison of Affymetrix signal levels between endothelial cells and bone biopsies

Probeset ID	Gene Symbol	Gene Title	p-value endothel vs. bone	Fold difference endothel vs. bone	Mean endothel signal level	Mean signal level (bone)
204719_at	ABCA8	ATP-binding cassette, sub-family A (ABC1), member 8	1.1×10^{-19}	-16.2	13	204
202502_at	ACADM	acyl-CoA dehydrogenase, C-4 to C-12 straight chain	2.3×10^{-6}	-1.9	227	440
201660_at	ACSL3	acyl-CoA synthetase long-chain family member 3	1.0×10^{-8}	1.8	676	376
201661_s_at	ACSL3	acyl-CoA synthetase long-chain family member 3	7.7×10^{-7}	2.0	487	250
201662_s_at	ACSL3	acyl-CoA synthetase long-chain family member 3	1.2×10^{-28}	3.7	515	138
205327_s_at	ACVR2A	activin A receptor, type IIA	2.4×10^{-4}	1.4	136	98
226192_at	AR	androgen receptor	1.6×10^{-4}	2.3	107	47
205950_s_at	CA1	carbonic anhydrase I	5.9×10^{-87}	-170.2	71	12006
210916_s_at	CD44	CD44 molecule (Indian blood group)	1.5×10^{-1}	-1.2	163	198
209967_s_at	CREM	cAMP responsive element modulator	5.9×10^{-39}	6.6	433	66
212888_at	DICER1	dicer 1, ribonuclease type III	2.3×10^{-10}	-1.7	405	669
205530_at	ETFDH	electron-transferring-flavoprotein dehydrogenase	3.5×10^{-6}	-1.7	75	130
205189_s_at	FANCC	Fanconi anemia, complementation group C	9.5×10^{-1}	1.0	73	73
210446_at	GATA1	GATA binding protein 1 (globin transcription factor 1)	3.9×10^{-32}	-8.4	58	488
1555590_a_at	GATA1	GATA binding protein 1 (globin transcription factor 1)	3.2×10^{-18}	-3.6	120	430
201007_at	HADHB	hydroxyacyl-CoA dehydrogenase/3-ketoacyl-CoA thiolase/enoyl-CoA hydratase (trifunctiona	1.4×10^{-2}	1.3	1964	1546
212642_s_at	HIVEP2	human immunodeficiency virus type I enhancer binding protein 2	8.7×10^{-1}	1.0	128	126
36004_at	IKBKG	inhibitor of kappa light polypeptide gene enhancer in B-cells, kinase gamma	1.5×10^{-1}	1.1	260	233
205992_s_at*	IL15	interleukin 15	5.7×10^{-3}	1.7	65	38
204686_at	IRS1	insulin receptor substrate 1	3.9×10^{-6}	-2.8	26	73
202351_at	ITGAV	integrin, alpha V	1.3×10^{-14}	4.9	1711	352
212274_at	LPIN1	lipin 1	2.4×10^{-4}	1.8	296	163
204041_at*	MAOB	monoamine oxidase B	1.6×10^{-8}	-3.4	44	149
205440_s_at	NPY1R	neuropeptide Y receptor Y1	1.1×10^{-3}	-2.1	7	15
205259_at	NR3C2	nuclear receptor subfamily 3, group C, member 2	1.2×10^{-11}	3.5	100	28
205301_s_at	OGG1	8-oxoguanine DNA glycosylase	2.6×10^{-1}	-1.1	78	86
200006_at	PARK7	parkinson protein 7	2.7×10^{-3}	-1.2	2376	2854
204491_at	PDE4D	phosphodiesterase 4D, cAMP-specific	5.5×10^{-3}	1.4	239	175
227088_at	PDE5A	phosphodiesterase 5A, cGMP-specific	4.4×10^{-18}	-5.0	33	164
223358_s_at	PDE7A	phosphodiesterase 7A	2.8×10^{-36}	-4.6	50	230
225207_at	PDK4	pyruvate dehydrogenase kinase, isozyme 4	4.3×10^{-3}	-2.1	323	693
223437_at	PPARA	peroxisome proliferator-activated receptor alpha	2.8×10^{-1}	1.1	231	203
232181_at	PPARGC1B	peroxisome proliferator-activated receptor gamma, coactivator 1 beta	6.1×10^{-1}	1.1	68	63
201408_at	PPP1CB	protein phosphatase 1, catalytic subunit, beta isozyme	4.5×10^{-8}	-2.2	199	442
202432_at	PPP3CB	protein phosphatase 3, catalytic subunit, beta isozyme	1.4×10^{-18}	-2.7	337	921
32541_at	PPP3CC	protein phosphatase 3, catalytic subunit, gamma isozyme	2.1×10^{-8}	2.1	128	62
211658_at	PRDX2	peroxiredoxin 2	5.3×10^{-14}	-2.3	516	1213
201006_at	PRDX2	peroxiredoxin 2	1.0×10^{-5}	-1.5	31	45
200845_s_at	PRDX6	peroxiredoxin 6	7.0×10^{-2}	1.1	1290	1134
201300_s_at	PRNP	prion protein	1.0×10^{-6}	1.8	1224	676
202716_at	PTPN1	protein tyrosine phosphatase, non-receptor type 1	1.6×10^{-2}	1.4	182	135
212610_at	PTPN11	protein tyrosine phosphatase, non-receptor type 11	1.5×10^{-2}	-1.2	1033	1201
223342_at	RRM2B	ribonucleotide reductase M2 B (TP53 inducible)	9.7×10^{-1}	1.0	127	127
203075_at	SMAD2	SMAD family member 2	6.3×10^{-5}	-1.4	292	403
204467_s_at	SNCA	synuclein, alpha (non A4 component of amyloid precursor)	1.9×10^{-26}	-7.9	294	2327
236081_at*	SNCA	synuclein, alpha (non A4 component of amyloid precursor)	1.2×10^{-19}	-7.4	67	494
219257_s_at	SPHK1	sphingosine kinase 1	1.1×10^{-24}	4.9	579	118
225252_at	SRXN1	sulfiredoxin 1	2.4×10^{-2}	1.3	440	337
211573_x_at	TGM2	transglutaminase 2	2.1×10^{-26}	5.5	383	69
202704_at	TOB1	transducer of ERBB2, 1	2.4×10^{-8}	-2.7	155	415
222919_at	TRDN	triadin	1.5×10^{-4}	-12.4	11	134
204712_at*	WIF1	WNT inhibitory factor 1	1.3×10^{-31}	-33.1	16	531
212637_s_at	WWP1	WW domain containing E3 ubiquitin protein ligase 1	5.1×10^{-1}	1.1	66	59

The table shows average Affymetrix signal levels from primary cultures of endothelial cells (n=3) or iliac bone biopsies (n=84). The analyses were run on the same chip type and data were normalized together. Probesets undetected in cardiomyocytes are marked with an asterix (*).

Table S4. Conditional FDR (<0.1) on SNPs from genes with BMD and CVD associated transcripts after controlling for multiple testing across phenotypes, location and effect

SNPs in genes with transcripts associated with femoral neck BMD

						Conjunctional FDR values								Location and effect of gene variant		
Locus #	SNP id	Gene id	pval_BMD	fdr_BMD	min_fdr	BMD_SBP	BMD_DBP	BMD_HDL	BMD_TG	BMD_T1D	BMD_T2D	BMD_LDL	BMD_BMI	Location	Most significantly affected transcription factor motif	Delta score
65	rs9309664	PPP1CB	7.5×10^{-6}	1.1×10^{-2}	5.6×10^{-3}	**5.6×10^{-3}**	**6.8×10^{-3}**	**6.4×10^{-3}**	**9.1×10^{-3}**	**7.4×10^{-3}**	**1.1×10^{-2}**	**1.1×10^{-2}**	**1.2×10^{-2}**	Intron	HDAC2_disc6	11.27
149	rs7670229	NR3C2	1.9×10^{-4}	1.1×10^{-1}	7.3×10^{-2}	**8.4×10^{-2}**	**7.3×10^{-2}**	1.1×10^{-1}	1.0×10^{-1}	NA	**7.6×10^{-2}**	1.1×10^{-1}	1.3×10^{-1}	Intron variant in ARHGAP10	-	-
159	rs7708952	PDE4D	7.9×10^{-4}	2.6×10^{-1}	8.7×10^{-2}	**8.7×10^{-2}**	2.2×10^{-1}	2.2×10^{-1}	1.7×10^{-1}	NA	2.4×10^{-1}	1.2×10^{-1}	2.6×10^{-1}	Intron	CEBPB_known4	-1.41
211	rs12526857	HIVEP2	8.1×10^{-5}	6.0×10^{-2}	3.9×10^{-2}	**4.7×10^{-2}**	**4.0×10^{-2}**	**3.9×10^{-2}**	**4.5×10^{-2}**	**6.2×10^{-2}**	**5.6×10^{-2}**	**5.6×10^{-2}**	**6.2×10^{-2}**	Intergenic	ELF1_known1/ PU.1_known2/SPIB	-11.97/-11.97/-5.54
376	rs11066320	PTPN11	4.9×10^{-4}	2.0×10^{-1}	1.7×10^{-2}	**2.4×10^{-2}**	**3.2×10^{-2}**	**5.5×10^{-2}**	1.5×10^{-1}	**1.7×10^{-2}**	1.6×10^{-1}	1.0×10^{-1}	1.1×10^{-1}	Intron	AP-1 disc_7/AP-4_1/ AP-4_3/E2A_3/Mrg_1	10.90/-11.23/ 11.48/3.32/3.47
460	rs6501769	ABCA8	1.0×10^{-3}	2.9×10^{-1}	6.5×10^{-2}	2.4×10^{-1}	2.2×10^{-1}	**6.5×10^{-2}**	2.3×10^{-1}	NA	2.6×10^{-1}	1.3×10^{-1}	3.1×10^{-1}	Intergenic variant	lrf_disc3	-0.34
507	rs6020562	PTPN1	1.1×10^{-3}	2.9×10^{-1}	7.4×10^{-2}	1.2×10^{-1}	**7.4×10^{-2}**	2.8×10^{-1}	2.5×10^{-1}	NA	2.4×10^{-1}	1.5×10^{-1}	2.8×10^{-1}	Regulatory region	GR_disc6/PRDMI_disc_1/ RXRA_known5	-0.85/-11.97/-1.00
						SNPs in genes with transcripts associated with lumbar spine BMD										
Locus #	SNP id	Gene id	pval_BMD	fdr_BMD	min_fdr	BMD_SBP	BMD_DBP	BMD_HDL	BMD_TG	BMD_T1D	BMD_T2D	BMD_LDL	BMD_BMI	Regulatory region	CACD_1/Ets_known1/ Foxd1_1/Foxf2/Foxi1	-2.58/-11.35/158/ 1.38/4.67
371	rs421224	FANCC	1.9×10^{-4}	1.0×10^{-1}	5.3×10^{-2}	**9.4×10^{-2}**	1.0×10^{-1}	**9.4×10^{-2}**	**9.1×10^{-2}**	NA	**5.3×10^{-2}**	1.0×10^{-1}	1.1×10^{-1}	Intergenic	Foxa_known4/Foxj1_1/ Foxj1_2/Foxk1	4.52/2.18/6.1/ 8.27
371	rs357547	FANCC	1.8×10^{-4}	1.0×10^{-1}	5.3×10^{-2}	**9.4×10^{-2}**	1.0×10^{-1}	**9.6×10^{-2}**	**9.1×10^{-2}**	NA	**5.3×10^{-2}**	1.0×10^{-1}	1.1×10^{-1}	Intergenic	-	-
443	rs12796719	CD44	1.7×10^{-4}	8.8×10^{-2}	4.9×10^{-2}	**8.5×10^{-2}**	**7.3×10^{-2}**	**8.1×10^{-2}**	**6.1×10^{-2}**	NA	**4.9×10^{-2}**	**8.6×10^{-2}**	**9.7×10^{-2}**	Intergenic	-	-
443	rs353597	CD44	1.3×10^{-4}	7.4×10^{-2}	5.2×10^{-2}	**7.4×10^{-2}**	**6.5×10^{-2}**	**7.0×10^{-2}**	**5.2×10^{-2}**	NA	**5.9×10^{-2}**	**5.8×10^{-2}**	**8.4×10^{-2}**	Intergenic	Nanog_disc2	-1.67
443	rs353591	CD44	1.6×10^{-4}	8.8×10^{-2}	5.0×10^{-2}	**6.9×10^{-2}**	**5.6×10^{-2}**	**8.3×10^{-2}**	**8.6×10^{-2}**	NA	**5.0×10^{-2}**	**8.6×10^{-2}**	**9.6×10^{-2}**	Regulatory region	MZF1::1-4_2/Spz1_1/ ZNF263_disc1	-1.87/1.11/ -7.81
443	rs4756190	CD44	2.4×10^{-4}	1.2×10^{-1}	7.2×10^{-2}	1.2×10^{-1}	1.1×10^{-1}	1.2×10^{-1}	**9.9×10^{-2}**	1.2×10^{-1}	**7.2×10^{-2}**	**7.3×10^{-2}**	1.2×10^{-1}	Intergenic	HNF4_disc1	11.86

Independent ($r2 < 0.2$) of SNP(s) with a conditional FDR < 0.1 (after Bonferoni correction for 8 traits) in Bone Mineral Density (BMD), given the significance level in the associated phenotype. We defined the most significant BMD SNP in each LD block based on the minimum conjunctional FDR for each associated phenotype. The most significant SNPs in each gene of the LD block are listed along with the associated phenotype that provided the signal. Conditional FDR values < 0.1, after adjusting for multiple testing across phenotypes are in bold. The following abbreviations were used: type 1 diabetes (T1D), type 2 diabetes (T2D), systolic blood pressure (SBP), diastolic blood pressure (DBP), high density lipoprotein (HDL), low density lipoprotein (LDL), triglycerides (TG), waist hip ratio (WHR), chromosome location (Map Loc.). NA indicates that a given SNP was not available for a given trait.

Data on location and effect of gene variant were obtained from the MSK-KP (https://msk.hugeamp.org/ "Delta score" indicates the difference between Reference and Alternate scores, representing the degree to which the TF motif is affected by the alternate allele.

Table S5. Verification of Affymetrix data with RNA sequencing

	Data from RNAseq				Data from Affymetrix analysis			
**Gene**	**Relative transcript levels (mean FPKM)**	**St. Dev**	**r**	**p-value**	**Affymetrix ID**	**Gene**	**BMD correlation Pearson r-values**	**p-value**
ABCA8	298	127	-0.28	1.15 X 10^{-2}	204719_at	ABCA8	-0.29	7.20 X 10^{-3}
ACADM	948	221	-0.36	1.10 X 10^{-3}	202502_at	ACADM	-0.30	6.30 X 10^{-3}
					201660_at	ACSL3	-0.45	1.58 X 10^{-5}
ACSL3	544	88	-0.23	4.40 X 10^{-2}	201661_s_at	ACSL3	-0.39	3.00 X 10^{-4}
					201662_s_at	ACSL3	-0.29	6.70 X 10^{-3}
ACVR2A	93	23	-0.40	3.00 X 10^{-4}	205327_s_at	ACVR2A	-0.29	7.70 X 10^{-3}
AR	63	31	-0.33	2.60 X 10^{-3}	226192_at	AR	-0.27	1.31 X 10^{-2}
CA1	37533	8666	0.34	1.80 X 10^{-3}	205950_s_at	CA1	0.27	1.41 X 10^{-2}
CD44	2684	394	0.16	1.47 X 10^{-1}	210916_s_at	CD44	0.28	9.30 X 10^{-3}
CREM	130	25	-0.03	7.96 X 10^{-1}	209967_s_at	CREM	-0.31	4.20 X 10^{-3}
DICER1	728	157	-0.25	2.59 X 10^{-2}	212888_at	DICER1	-0.30	5.90 X 10^{-3}
ETFDH	522	92	-0.27	1.57 X 10^{-2}	205530_at	ETFDH	-0.29	7.50 X 10^{-3}
FANCC	173	28	0.08	4.87 X 10^{-1}	205189_s_at	FANCC	0.33	2.20 X 10^{-3}
GATA1	1857	368	0.21	6.54 X 10^{-2}	210446_at	GATA1	0.30	6.20 X 10^{-3}
					1555590_a_at	GATA1	0.29	8.00 X 10^{-3}
HADHB	2286	507	-0.30	6.90 X 10^{-3}	201007_at	HADHB	-0.32	3.10 X 10^{-3}
HIVEP2	228	52	-0.25	2.51 X 10^{-2}	212642_s_at	HIVEP2	-0.28	9.80 X 10^{-3}
IKBKG	Undetected	NA	NA	NA	36004_at	IKBKG	0.29	7.10 X 10^{-3}
IL15	56	13	-0.20	7.74 X 10^{-2}	205992_s_at	IL15	-0.29	6.80 X 10^{-3}
IRS1	102	45	-0.34	1.70 X 10^{-3}	204686_at	IRS1	-0.34	1.60 X 10^{-3}
ITGAV	344	124	-0.23	4.18 X 10^{-2}	202351_at	ITGAV	-0.28	9.60 X 10^{-3}
MAOB	224	109	-0.34	1.90 X 10^{-3}	204041_at	MAOB	-0.32	2.80 X 10^{-3}
NPY1R	57	34	-0.27	1.54 X 10^{-2}	205440_s_at	NPY1R	-0.28	1.08 X 10^{-2}
NR3C2	57	20	-0.34	2.00 X 10^{-3}	205259_at	NR3C2	-0.30	6.30 X 10^{-3}
OGG1	510	82	0.12	3.01 X 10^{-1}	205301_s_at	OGG1	-0.27	1.33 X 10^{-2}
PARK7	1741	252	-0.11	3.47 X 10^{-1}	200006_at	PARK7	-0.27	1.36 X 10^{-2}
PDE4D	310	61	-0.26	2.01 X 10^{-2}	204491_at	PDE4D	-0.28	1.06 X 10^{-2}
PDE5A	315	87	-0.32	3.60 X 10^{-3}	227088_at	PDE5A	-0.31	4.20 X 10^{-3}
PDE7A	824	116	-0.26	1.84 X 10^{-2}	223358_s_at	PDE7A	-0.29	7.10 X 10^{-3}
PDK4	1405	735	-0.34	1.70 X 10^{-3}	225207_at	PDK4	-0.34	1.70 X 10^{-3}
PPARA	508	102	-0.21	5.90 X 10^{-2}	223437_at	PPARA	-0.34	1.70 X 10^{-3}
PPARGC1B	109	24	-0.30	7.60 X 10^{-3}	232181_at	PPARGC1B	-0.35	9.00 X 10^{-4}
PPP1CB	2317	424	-0.32	4.10 X 10^{-3}	201408_at	PPP1CB	-0.30	5.70 X 10^{-3}
PPP3CB	931	175	-0.42	1.00 X 10^{-4}	202432_at	PPP3CB	-0.39	3.00 X 10^{-4}
PPP3CC	225	42	-0.36	1.20 X 10^{-3}	32541_at	PPP3CC	-0.33	2.40 X 10^{-3}
PRDX2	10252	1968	0.30	7.00 X 10^{-3}	211658_at	PRDX2	0.37	5.00 X 10^{-4}
					201006_at	PRDX2	0.27	1.18 X 10^{-2}
PRDX6	3284	493	-0.04	6.95 X 10^{-1}	200845_s_at	PRDX6	-0.34	1.80 X 10^{-3}
PTPN1	1053	153	0.01	9.19 X 10^{-1}	202716_at	PTPN1	0.35	1.30 X 10^{-3}
PTPN11	1227	244	-0.25	2.27 X 10^{-2}	212610_at	PTPN11	-0.32	3.30 X 10^{-3}
RRM2B	306	62	-0.27	1.40 X 10^{-2}	223342_at	RRM2B	-0.30	1.06 X 10^{-2}
SMAD2	880	108	-0.12	2.81 X 10^{-1}	203075_at	SMAD2	-0.29	6.50 X 10^{-3}
SNCA	6229	1387	0.24	3.41 X 10^{-2}	204467_s_at	SNCA	0.30	5.40 X 10^{-3}
					236081_at	SNCA	0.34	1.60 X 10^{-3}
SPHK1	274	53	0.18	1.12 X 10^{-1}	219257_s_at	SPHK1	0.28	1.13 X 10^{-2}
SRXN1	Undetected	NA	NA	NA	225252_at	SRXN1	0.29	7.90 X 10^{-3}
TGM2	1442	238	-0.02	8.82 X 10^{-1}	211573_x_at	TGM2	0.31	3.70 X 10^{-3}
TOB1	307	72	-0.31	4.80 X 10^{-3}	202704_at	TOB1	-0.28	9.30 X 10^{-3}
TRDN	805	1025	-0.36	1.20 X 10^{-3}	222919_at	TRDN	-0.28	1.07 X 10^{-2}
WIF1	551	257	0.38	6.00 X 10^{-4}	204712_at	WIF1	0.37	5.00 X 10^{-4}
WWP1	656	158	-0.41	2.00 X 10^{-4}	212637_s_at	WWP1	-0.37	6.00 X 10^{-4}

RNAseq data were FPKM transformed by correcting for total read count per sample and transcript length using the following formula: (10^9 * Counts)/(total reads * length of transcript) All transcripts with a FPKM >= 1 in at least 10 samples were retained, rest discarded. BMD correlation refers to total hip BMI adjusted Z-scores (age and body mass index adjusted BMD). The second column displays Pearson correlation coefficients (r) between indicated transcripts and total hip BMI adjusted Z-score.

Table S6. Associations between Affymetrix signal values, RNAseq reads, and PCR ddCT values

Gene	Affymetrix vs RNAseq	Affymetrix vs PCR	RNAseq vs PCR
**SOST**	0.871	0.889	0.839
**ACSL3**	0.265	-0.122	0.257
**ELTD1**	Undetected by RNAseq	0.148	Undetected by RNAseq
**COPS4**	0.171	-0.079	0.068
**MEPE**	0.894	0.758	0.767
**MMP13**	0.839	0.858	0.786
**SFRP4**	0.892	0.733	0.808
**DKK1**	0.855	0.811	0.806
**CHAD**	0.808	0.796	0.760
**SOX4**	0.661	0.221	0.328
**RUNX2**	0.570	-0.116	0.216
**LMOD3**	0.773	0.779	0.672
**DMD**	0.872	0.485	0.502
**NFIB**	0.705	0.397	0.448
**EFEMP1**	0.876	0.687	0.673
**ASB5**	0.635	0.866	0.614
**PHEX**	0.814	0.615	0.558
**CAP2**	0.734	0.904	0.735
**PKIA**	0.861	0.912	0.809
**CLIC5**	0.714	0.763	0.690

The table contains Pearson correlation r-values of indicated transcripts between pairs of Affymetrix signal values, RNAseq reads, or PCR ddCT values obtained from 80 iliac bone biopsies. Shaded cells indicate highest correlation value for each gene. There are in general a high degree of concordance between results using the three technologies, Affymetrix analysis vs RNAseq shows best reproduction.

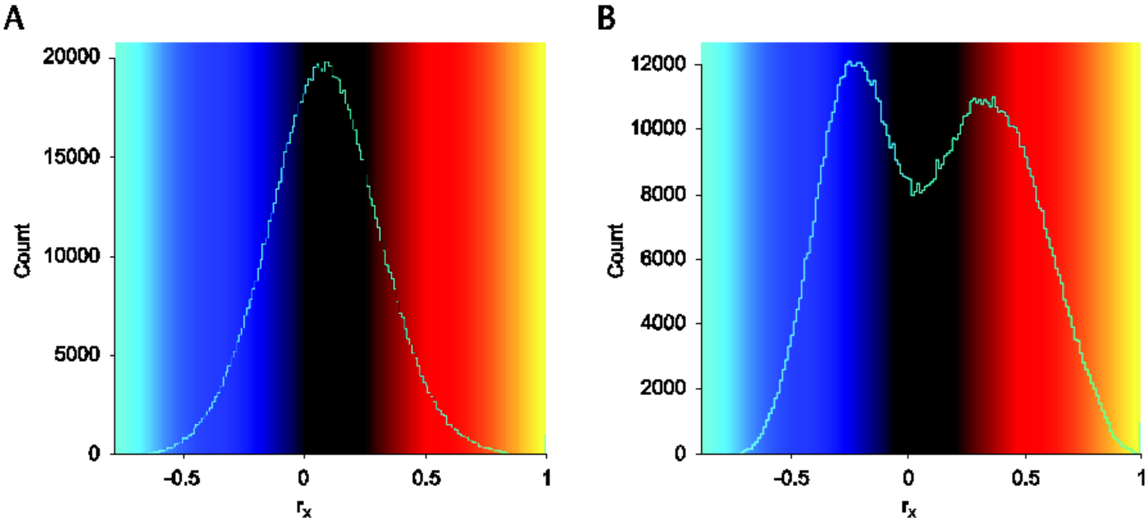

Figure S1
A: When a cluster matrix was made from the 1000 mRNAs and miRNAs with lowest correlation to BMD, a single peak around the zero rx-value appeared when plotting Pearson correlation rx-values against the number of correlations. The transcripts which correlated positively to each other are shown in red and yellow colors, and negative correlations in bluish colors. **B:** Plotting the 1000 genes with highest correlation to BMD resulted in 2 peaks, each appearing asymmetrical with maxima clearly off the 0-value, suggesting non-random results with distributions as positive or negative correlations.
